# Topophilia and Quality of Life: Defining the Ultimate Restorative Environment

**Published:** 2005-02

**Authors:** Hakon Heimer

Mental stress is increasingly recognized as an environmental contributor to disease burden in many parts of the world. One way people try to reduce mental stress is through features of the built environment. Architects often give us “nature” in the form of water or trees to create restorative settings, while others favor complex and challenging sculpture or structures. In this month’s issue, Oladele A. Ogunseitan of the University of California, Irvine, asks us to consider the study of topophilia as a source for criteria to help us judge which elements of an environment truly have a restorative effect **[*EHP* 113:143–148]**.

The term *topophilia* was coined by the geographer Yi-Fu Tuan of the University of Wisconsin and is defined as the affective bond with one’s environment—a person’s mental, emotional, and cognitive ties to a place. Topophilia is studied here as a latent construct, an abstract psychological concept similar to “attitude” or “intelligence” whose variability can only be observed indirectly through its effect on measurable responses.

Ogunseitan surveyed 379 people on the Irvine campus, asking them to rate the importance of features such as color, flowers, and complexity to an environment they consider restorative. He then compared these values with data from a World Health Organization quality of life (QOL) survey administered along with the topophilia questions.

The environmental features surveyed mapped closely onto four domains of topophilia: ecodiversity (the presence of flowers, water, and other elements of nature), synesthetic tendency (a commingling of colors, smells, and other sensory stimuli), environmental familiarity (which includes spaciousness and privacy), and cognitive challenge (which includes structural complexity and texture). Structural equation modeling showed a positive correlation between topophilia and various aspects of QOL. That is, people who had the highest topophilia ratings (who, for example, most highly valued flowers or color as important for achieving a restorative effect) tended to have the highest QOL scores. Ecodiversity had the highest correlation with overall QOL. Within this category, the presence of flowers and proximity to lakes or the ocean were most significantly correlated with QOL.

Ogunseitan notes that “complexity” and other features associated with the cognitive domain—and much loved by some architects—were not linked with higher QOL. He also was surprised that none of the synesthetic tendency qualities such as smells or sounds were associated with improved QOL, given the postulated health benefits—and obvious commercial appeal—of aromatherapy and recorded “nature” sounds.

The results would appear to buttress previous research indicating that people prefer “natural” environments to those that emphasize complex designs or artificial sensory stimulation, but Ogunseitan stresses that this study represents only an initial foray into understanding the complex relationship between quality of life and restorative environments. The current study design assumes that when people rate aspects of their environment highly, there really is a chance that those features are restorative to mental health beyond their aesthetic appeal. Although the results of such correlational research do not imply causation, Ogunseitan notes, they can offer insights to guide future research.

